# Develop and validate a fair machine learning model to identify patients with high data-continuity in electronic health records data

**DOI:** 10.1093/jamiaopen/ooag124

**Published:** 2026-07-13

**Authors:** Yao An Lee, Tiange Tang, Yu Huang, Jiang Bian, Lizheng Shi, Jingchuan Guo

**Affiliations:** Center for Biomedical Informatics, Regenstrief Institute, Indianapolis, IN 46202, United States; Department of Health Policy and Management, Celia Scott Weatherhead School of Public Health and Tropical Medicine, Tulane University, New Orleans, LA 70112, United States; Center for Biomedical Informatics, Regenstrief Institute, Indianapolis, IN 46202, United States; Department of Biostatistics & Health Data Science, Indiana University, Indianapolis, IN 46202, United States; Center for Biomedical Informatics, Regenstrief Institute, Indianapolis, IN 46202, United States; Department of Biostatistics & Health Data Science, Indiana University, Indianapolis, IN 46202, United States; Department of Health Policy and Management, Celia Scott Weatherhead School of Public Health and Tropical Medicine, Tulane University, New Orleans, LA 70112, United States; Center of Health Systems Analytics Research, Tulane University, New Orleans, LA 70112, United States; Center for Biomedical Informatics, Regenstrief Institute, Indianapolis, IN 46202, United States; Department of Pharmacy Practice, Purdue University College of Pharmacy, Indianapolis, IN 46202, United States

**Keywords:** EHR, data continuity, machine learning prediction

## Abstract

**Objectives:**

Electronic health record (EHR) data discontinuity, defined as receiving care outside of a particular EHR system, may cause misclassification of study variables. We aimed to: (1) quantify misclassification across levels of EHR data discontinuity and identify an optimal continuity threshold, (2) develop a machine learning (ML) model to predict EHR continuity and optimize fairness across racial and ethnic groups, and (3) externally validate the EHR continuity prediction model using an independent dataset.

**Materials and Methods:**

We used linked OneFlorida+ EHR-Medicaid claims data for model development and Research Action for Health Network (REACHnet) EHR-Louisiana Blue Cross Blue Shield (LABlue) claims data for external validation. A Harmonized Encounter Proportion Score (HEPS), adapted from prior continuity metrics, was applied to quantify patient-level EHR data continuity and the impact on misclassification of 42 clinical variables. Machine learning models were trained using routinely available demographic, clinical, and healthcare utilization features derived from structured EHR data.

**Results:**

Higher EHR data continuity was associated with lower rates of misclassification. A HEPS threshold of approximately 30% effectively distinguished patients with sufficient data continuity. Machine learning models demonstrated strong performance in predicting high continuity (area under the receiver operating characteristic curve [AUROC] = 0.77). Fairness assessments showed bias against Hispanic group, which was substantially improved following bias mitigation procedures. Model performance remained robust and fair in the external validation.

**Discussion:**

Our study offers a practical metric for quantifying data continuity in EHR networks. The current ML model incorporating EHR-routinely collected information can accurately identify patients with high care continuity.

**Conclusions:**

We developed a generalizable data-continuity classification tool that can be easily applied across EHR systems, strengthening the rigor of EHR-based research.

## Introduction

Electronic health records (EHRs) have become a cornerstone of real-world data (RWD), offering a wealth of clinical information that is increasingly utilized for research purposes. Among these studies, EHRs are particularly valuable in comparative effectiveness research (CER) because of their rich clinical detail, longitudinal structure, and growing accessibility through national research networks.^1^^,^^2^ National network, such as Patient-Centered Clinical Research Network (PCORnet), is a “network of networks” supported by the Patient-Centered Outcomes Research Institute (PCORI)^3^^,^^4^ and has enabled access to population-scale EHR data covering over 70 million patients across the United States.^5^ However, a major limitation of EHR-based RWD is the fragmentation of the US healthcare system, where patients often receive care across multiple, unlinked providers, and most EHR systems do not capture care delivered outside their affiliated settings. This discontinuity is further compounded by variability in healthcare access, driven in part by social determinants of health,^6^^,^^7^ which influence patients’ patterns of care-seeking across different systems. As a result, medical information recorded in other healthcare systems often remains “invisible” to investigators, leading to low EHR continuity, or EHR data discontinuity. These challenges contribute to EHR data discontinuity, which in this study refers to care received outside the indexed EHR system and therefore not captured in that system’s records, posing a substantial risk of introducing information bias in research studies.^8^^,^^9^ Studies have shown that patients with low EHR continuity experience up to a 17-fold higher misclassification in key variables compared to those with high continuity.^10^

Without linkage to additional data sources, researchers face significant limitations in assessing the completeness of patient records, leaving their analyses vulnerable to potential misclassification of exposures, outcomes, and key covariates used in confounding control. In contrast, insurance claims data offer comprehensive capture of healthcare utilization within defined enrollment periods, although they often lack the clinical nuance present in EHRs.^11^ While combining EHRs with claims data can substantially enhance data completeness and reliability, such linkages remain uncommon due to challenges related to privacy regulations, data governance, and technical feasibility. These limitations highlight the pressing need to systematically measure EHR continuity and to establish scalable, transparent approaches for mitigating its influence on data integrity and the validity of real-world evidence generated from EHR-based studies. Patients with high EHR data continuity, where most clinical encounters are captured within a single EHR system, provide a more reliable foundation for clinical research and decision-making. Conversely, low EHR data continuity, characterized by fragmented records across disparate systems, can obscure important clinical information and exacerbate inequities in research and care.^6^

At the same time, while restricting CER cohorts to patients with high EHR continuity can improve validity, doing so without accounting for structural inequities may unintentionally exclude underserved populations, particularly racial and ethnic minorities with fewer care options.^12^ Evidence shows that machine learning (ML) systems trained on incomplete or biased data can perpetuate or amplify existing health disparities by underestimating care needs for Black patients when cost is used as a proxy for health status, or by failing to detect clinical signs in racial groups underrepresented in training datasets.^13^ As a result, there is increasing urgency to assess and ensure fairness in ML-driven healthcare tools. This includes evaluating algorithmic behavior using fairness metrics such as demographic parity, equalized odds, and predictive rate parity,^14–17^ all of which aim to prevent systematic disadvantages across protected subgroups. Despite the growing literature, there remains no consensus on which fairness metrics should be applied in different contexts, highlighting the need for careful selection and application based on the specific characteristics and implications of the healthcare setting.

Prior work has shown that EHR data continuity or completeness can be assessed using linked reference data and predicted using EHR-only features. Lin et al^8–10^ established an important framework for continuity measurement, misclassification assessment, and EHR-based prediction, and Klann et al^18^ later described a multisite “loyalty/completeness” approach for identifying patients with more complete EHR data. Building on this prior work, our study refines the continuity metric for linked EHR-claims settings, evaluates broader EHR predictor sets beyond predefined proxies, and extends the framework by relating continuity to downstream misclassification thresholding and fairness-aware prediction.

## Objective

The objective of this study was to improve the validity of EHR data and promote health equity by developing and validating a ML algorithm to identify patients with high EHR data continuity. Specifically, we (1) evaluated how EHR data discontinuity contributes to misclassification bias and identified an optimal Harmonized Encounter Proportion Score (HEPS) threshold for acceptable data completeness in our EHR network; (2) developed a ML algorithm to predict EHR data continuity at the person level and assessed algorithmic fairness across racial and ethnic subgroups; and (3) externally validated the continuity prediction algorithm in an independent EHR dataset. Using linked EHR-Florida Medicaid claims data from OneFlorida+ network, we assessed the degree of misclassification across varying levels of data continuity to establish a threshold at which EHRs alone are considered sufficiently complete for valid inference. Furthermore, to ensure transparency and equity, we incorporated model explainability tool and applied fairness metrics to evaluate and mitigate disparities in model performance across racial and ethnic groups. Finally, we validated our continuity prediction algorithm in linked EHR-claims data from Research Action for Health Network (REACHnet) EHRs and Louisiana Blue (LABlue) claims network to evaluate generalizability and transportability. Collectively, this work sought to deliver a tool that improves research validity through rigorous identification of patients with adequate data continuity and guarantees equitable performance across heterogeneous populations and healthcare systems.

## Materials and methods

### Data source

The study used 4 underlying data sources organized into 2 linked analytic datasets: (1) OneFlorida+ EHR linked at the patient level to Florida Medicaid claims for model development and internal validation, and (2) REACHnet EHR linked at the patient level to LABlue commercial claims for external validation. OneFlorida+ and REACHnet are members of the National Patient-Centered Clinical Research Network (PCORnet)^19^ and follow the PCORnet Common Data Model (CDM)^5^ that provide longitudinal structured EHR data, including demographics, diagnoses, vital signs, and healthcare utilization within each system. Florida Medicaid and LABlue claims provide longitudinal records of reimbursed healthcare utilization during enrollment and were used as reference standards to quantify the magnitude of data continuity on the EHR side.

### Study design and population

Using these linked datasets, we identified patients in OneFlorida+ between January 2015 and October 2023 and in REACHnet between January 2022 and September 2024. Eligible patients from both networks met predefined criteria: (1) adults aged 18 or older and (2) had at least 1 encounter recorded in the EHR system during their active enrollment period. We excluded individuals who (1) had fewer than 12 months of EHR or claims data, or (2) had a secondary payer.

**Figure 1.**
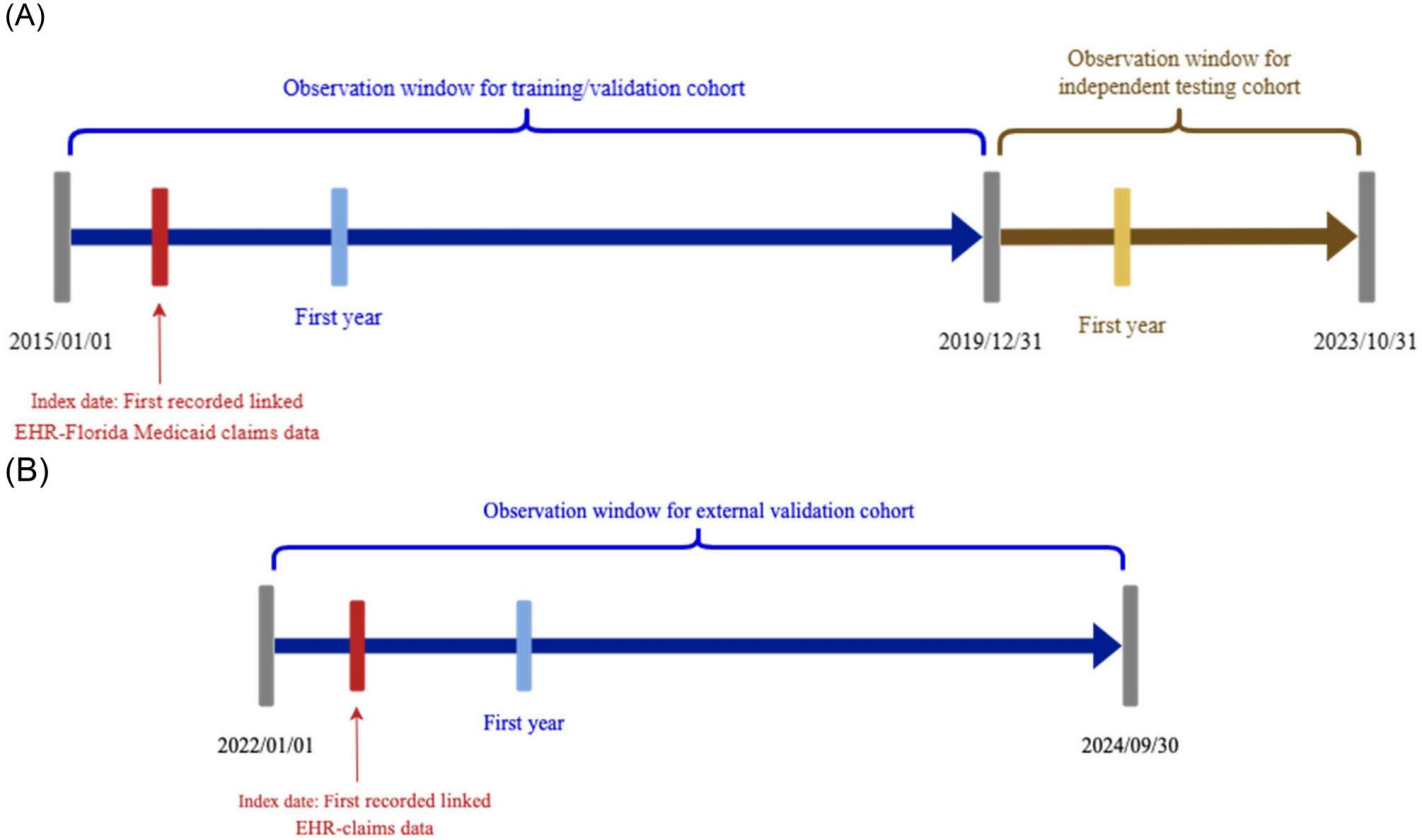
Overview of study design for (A) OneFlorida+ and (B) REACHnet-LABlue. Panel A shows the study design and data linkage for the OneFlorida+ electronic health record (EHR)-Medicaid claims cohort used for model development. Panel B shows the corresponding design and EHR-Louisiana Blue Cross Blue Shield (LABlue) claims linkage for the REACHnet cohort used for external validation of the continuity prediction model.

For each included patient, the date of the first recorded instance of linked EHR-claims data within the study period was designated as the index date (Figure 1), and patients were followed for 1 year thereafter to evaluate data continuity. In OneFlorida+, patients with a first complete year of data between 2015 and 2019 were used as the training/internal-validation cohort, whereas the cohort identified during 2020-2023 was used for independent testing. In the REACHnet-LABlue data, patients with a first complete year of data during 2022-2024 were used as the external-validation cohort.

### EHR data-continuity

To identify the optimal level of EHR data continuity, we applied the HEPS,^20^ a continuity metric developed to robustly estimate data completeness at the patient-level. Harmonized Encounter Proportion Score ranges from 0% to 100%, where higher values indicate a greater proportion of observable encounters captured in the EHR relative to linked claims, and lower values indicate more incomplete EHR capture. Harmonized Encounter Proportion Score is adapted from the Mean Proportion of Encounters Captured (MPEC)^8^^,^^10^ algorithm. The MPEC algorithm calculates the proportion of healthcare encounters documented in the EHR compared to claims data:


$$\mathrm{MPEC}=\;(\frac{\mathrm{Outpatient}\;\mathrm{encouters}\;in\;\mathrm{EHRs}}{\mathrm{Oupatient}\;\mathrm{encouters}\;in\;cl\mathrm{aims}}\;+\;\frac{\mathrm{Inpatient}\;\mathrm{encouters}\;in\;\mathrm{EHRs}}{\mathrm{Inaptient}\;\mathrm{encouters}\;in\;\mathrm{claims}})/2$$

While MPEC is informative, MPEC becomes undefined when patients have no encounters in either the inpatient or outpatient setting during the observation period, resulting in a zero denominator. Under prior formulations using multiple encounter categories, patients with utilization concentrated in a single setting may receive lower or undefined continuity scores, where the absent encounter type reflects underlying care patterns rather than data incompleteness. Harmonized Encounter Proportion Score addresses this limitation by recursively handling absent encounter types, allowing continuity to be estimated even when utilization is concentrated in a single setting. By reducing over-penalization of such single-encounter-type utilization patterns, HEPS provides a more robust and interpretable patient-level continuity measure in heterogeneous EHR-claims settings.

The HEPS formula is defined as follows:


HEPS(OC,OE,IC,IE)={(OEOC+IEIC)/2,if OEOC≥0 and IEIC≥0HEPSO(OC, OE, IC, IE),otherwise

HEPSO(OC,OE,IC,IE)={OEOC,if OC>0 and IC≤0HEPSI(OC, OE, IC, IE),otherwise

HEPSI(OC,OE,IC,IE)={IEIC,if IC>0 and OC≤0-1,otherwise

OE: outpatient encounters in EHRs; IE: inpatient encounters in EHRs

OC: outpatient encounters in claims; IC: inpatient encounters in claims

Using HEPS, we evaluated magnitude of EHR data continuity for each patient. We restricted the analytic cohort to patients with HEPS > 0% during the 1-year observation window, because HEPS ≤ 0% indicates no captured inpatient and outpatient encounters in the EHR, the linked claims, or both. Such cases provide insufficient clinical information for continuity assessment and model development; therefore, they were excluded from the final analysis.

### Misclassification tests and evaluation metrics

To identify an optimal HEPS cutoff for defining high vs low EHR continuity, we adopted a methodology based on the framework proposed by Lin et al.^8–10^ We performed misclassification analyses using 2 types of clinical information commonly used in observational research:

1. Comorbidity indices:
  - Charlson Comorbidity Index (CCI) (range: 0 to 35),
  - Elixhauser Comorbidity Index (ECI) (range: −19 to 89), and
  - A combined score integrating both indices (range: −2 to 26), where higher scores are associated with increased mortality risk.^21^ This combined score is a validated composite measure derived from the Charlson and Elixhauser indices and was included to provide a broader summary of comorbidity burden than either index alone.
2. Clinical variables: A set of 42 variables was assessed, comprising 30 chronic conditions,^22^ and 12 frequently prescribed medications (see full list in Table S1; definitions in Table S2). These variables were evaluated during each patient’s first complete calendar year of data.

For each patient, 2 comorbidity scores were calculated separately using EHR-only data and linked EHR-claims data. The difference between the 2 (ie, score_full-score_EHR) represents the extent to which comorbidity burden may be underestimated when using EHR data alone compared with using both claims and EHRs. We then compared the average differences across HEPS strata to evaluate the relationship between data continuity and score accuracy.

For the 42 selected clinical variables, we further assessed covariate balance between EHR-only data and linked EHR-claims data across HEPS deciles using 2 metrics: mean standardized differences (MSDs) and sensitivity. The MSD compared classifications based on EHR-only data vs linked claims-EHR data; it quantified the difference between the 2 group means, standardized by their pooled standard deviation, and is commonly employed to evaluate covariate balance between comparison groups.^23^ A smaller MSD suggested a stronger agreement between data sources, indicating that the EHR alone provides sufficient information to approximate classifications derived from the more complete, linked dataset. Sensitivity of EHR coverage was defined as the proportion of patients identified with a given variable using EHR data alone, relative to those identified using the linked claims-EHR data:


$${\mathrm{Sen}t\mathrm{ivity}}_{\mathrm{variable}(i)}=\frac{No.\;of\;\mathrm{patients}\;\mathrm{with}\;\mathrm{variable}(i)=1\;\mathrm{based}\;on\;\mathrm{EHRs}\;\mathrm{alone}}{No.\;of\;\mathrm{patients}\;\mathrm{with}\;\mathrm{variable}(i)=1\;\mathrm{based}\;on\;\mathrm{linked}\;\mathrm{claims}-\mathrm{EHRs}},\;i=1-42$$

Given that the reference standard suggests classification based on all available data (both claims and EHR data), specificity was assumed to be 100%. However, incomplete coding in the EHR could result in reduced sensitivity, signaling potential misclassification.

Based on prior literature, a standardized difference < 0.10 is generally considered indicative of acceptable covariate balance and minimal confounding.^24^^,^^25^ A threshold of less than 0.10 for standardized differences across all variables was determined to indicate satisfactory balance of covariates in the context of achieving adequate confounding adjustment. Thus, we selected the HEPS threshold that achieved standardized differences below this benchmark across all 42 variables, defining this point as the optimal study-specific cutoff for high EHR data continuity.

### Model development, explainable AI, and fairness optimization

To predict EHR data continuity, we identified a comprehensive set of candidate predictors based on clinical expertise^8^ and existing literature.^20^ Lin et al^8^ proposed a list of candidate proxy indicators including (a) general examinations or routine care; (b) preventive interventions; (c) recording of diagnoses or medications in the EHR; (d) having a certain type and number of visits in the EHR; and (e) seeing the same provider repeatedly within the system (see Table S3 for detailed definitions). We further incorporated demographic, vital signs, and clinical diagnoses variables from Huang et al,^20^ including common diseases and provider-related factors. These predictors encompassed sociodemographic characteristics, vital signs, healthcare utilization patterns, and clinical indicators. The output variable was a binary indicator of high or low data continuity, as defined by the HEPS cutoff identified earlier.

To evaluate and optimize predictive performance and feasibility, we developed models using the predictor set identified from Huang et al (predictor set 1), the predictor set identified from Lin et al (predictor set 2), and the complete (combined) predictor set. Both logistic regression (LR) and XGBoost^26^ algorithms were used to develop prediction models. For each patient, the first complete calendar year of data following the index date (Figure 1A) was extracted for model development. Within the development period, these data were randomly split into training (70%) and validation (30%) cohorts, whereas the later temporal cohort was used for independent testing. Model performance was assessed using standard classification metrics: area under the receiver operating characteristic curve (AUROC), F1 score, precision, and recall.

Model interpretability was enhanced using explainable AI techniques, specifically SHAP (SHapley Additive exPlanations)^27^ values, to identify key predictors influencing EHR continuity. Additionally, 7 ML fairness metrics were applied to evaluate model fairness across racial and ethnic groups: non-Hispanic Black (NHB) vs non-Hispanic White (NHW), and Hispanic vs NHW. In this study, the protected group refers to the subgroup of primary equity interest (NHB or Hispanic), and the privileged group refers to the reference subgroup (NHW). We selected the false-negative rate (FNR)—the proportion of high-continuity patients incorrectly classified as having low continuity—as the primary fairness target because, in this use case, false negatives lead to the inappropriate exclusion of patients who actually have adequate EHR continuity, thereby reducing cohort representativeness and potentially introducing systematic underrepresentation of certain groups. This choice reflects a use-case-specific priority to minimize unjust exclusion, while acknowledging the trade-off that optimizing FNR alone may increase false positives and reduce balance on other fairness or overall performance metrics.

### External validation

Using the models developed from OneFlorida+ data, we assessed their generalizability in a linked EHR-claims dataset from the REACHnet-LABlue network. First, we replicated the misclassification analyses by (1) comparing mean differences in 3 comorbidity indices—CCI, ECI, and combined score—across HEPS strata to identify the minimal HEPS needed to achieve acceptable classification; and (2) evaluating covariate balance across 42 selected variables (30 chronic conditions and 12 commonly prescribed medications) using MSD, with < 0.10 across all variables indicating satisfactory balance. Second, we evaluated transportability by applying the OneFlorida+-trained LR and XGBoost models to the REACHnet-LABlue predictors without recalibration. Model performance was evaluated across prespecified predictor sets (predictor set 1; predictor set 2) and the complete predictor set.

## Results

A total of 5462 OneFlorida+ adults with a complete first calendar year (2015-2019) and HEPS > 0% were included in the final analytic cohort; an additional 3364 patients with a complete first calendar year (2020-2023) and HEPS > 0% comprised the testing cohort. In the REACHnet-LABlue system, 14 567 adults met eligibility criteria and were included in the external validation cohort. Table 1 summarizes baseline characteristics. The OneFlorida+ training and testing cohorts were older than the REACHnet cohort (mean age 47.03 and 48.49 years, respectively, vs 45.14 years in REACHnet). The female proportion (∼65%-67%) was similar across cohorts. Racial/ethnic composition differed: OneFlorida+ included larger Hispanic (25%-26%) and NHB (29%-31%) groups compared with the REACHnet cohort. Detailed provider-type and laboratory distributions are shown in Table S4.

**Table 1.** Population characteristics of training, testing, and validation cohort.

Variable	OneFlorida+		REACHnet-LABlue
	Training cohort, N (%)	Testing cohort, N (%)	Validation cohort, N (%)
Total	5462 (100.0)%	3364 (100.0)%	14567 (100.0)%
AGE_AT_INDEX, years, mean (SD)	47.03 (15.61)	48.49 (14.2)	45.14 (13.16)
BMI			
Mean (SD)	31.48 (8.94)	32.16 (9.04)	29.55 (7.11)
BMI_missing	973 (17.81)%	587 (17.45)%	1389 (9.54)%
Blood pressure			
Systolic blood pressure, mean (SD)	128.42 (19.36)	130.12 (19.6)	124.67 (13.82)
Systolic blood pressure_missing	1075 (19.68)%	655 (19.47)%	1535 (10.54)%
Diastolic blood pressure, mean (SD)	76.3 (11.86)	77.41 (11.56)	77.7 (8.66)
Diastolic blood pressure_missing	1074 (19.66)%	655 (19.47)%	1535 (10.54)%
Race_Ethnicity			
Hispanics	1437 (26.31)%	855 (25.42)%	491 (3.37)%
NHB	1706 (31.23)%	1005 (29.88)%	2256 (15.49)%
NHW	2159 (39.53)%	1382 (41.08)%	11408 (78.31)%
Others	43 (0.79)%	33 (0.98)%	251 (1.72)%
Unknown	117 (2.14)%	89 (2.65)%	161 (1.11)%
SEX			
SEX_F	3649 (66.81)%	2232 (66.35)%	9437 (64.78)%
Smoking_Status			
Current smoker	946 (17.32)%	519 (15.43)%	626 (4.3)%
Former smoker	653 (11.96)%	521 (15.49)%	736 (5.05)%
Never smoker	1246 (22.81)%	790 (23.48)%	11808 (81.06)%
Unknown	2617 (47.91)%	1534 (45.6)%	1397 (9.59)%
Common and CCI disease			
Obesity	1273 (23.31)%	950 (28.24)%	2161 (14.83)%
Hypertension	2361 (43.23)%	1543 (45.87)%	1957 (13.43)%
Stroke/transient ischemic attack	183 (3.35)%	111 (3.3)%	70 (0.48)%
Myocardial infarction	215 (3.94)%	125 (3.72)%	59 (0.41)%
Ischemic heart disease	488 (8.93)%	307 (9.13)%	244 (1.68)%
Family history of diabetes	288 (5.27)%	126 (3.75)%	60 (0.41)%
Alcohol use disorder	219 (4.01)%	120 (3.57)%	65 (0.45)%
Type 2 diabetes (T2D)	1806 (33.06)%	1277 (37.96)%	715 (4.91)%
Acute myocardial infarction	69 (1.26)%	46 (1.37)%	31 (0.21)%
Congestive heart failure	421 (7.71)%	296 (8.8)%	95 (0.65)%
Peripheral vascular disorder	407 (7.45)%	280 (8.32)%	198 (1.36)%
Cerebrovascular disease	338 (6.19)%	203 (6.03)%	133 (0.91)%
Dementia	48 (0.88)%	28 (0.83)%	8 (0.05)%
Chronic pulmonary disease	1040 (19.04)%	605 (17.98)%	751 (5.16)%
Rheumatoid arthritis/collagen vascular diseases	239 (4.38)%	188 (5.59)%	946 (6.49)%
Ulcer disease	63 (1.15)%	37 (1.1)%	40 (0.27)%
Mild liver disease	434 (7.95)%	285 (8.47)%	394 (2.7)%
Uncomplicated diabetes	1555 (28.47)%	1008 (29.96)%	550 (3.78)%
Complicated diabetes	957 (17.52)%	851 (25.3)%	429 (2.95)%
Hemiplegia or paraplegia	127 (2.33)%	58 (1.72)%	29 (0.2)%
Renal failure	425 (7.78)%	293 (8.71)%	177 (1.22)%
Any tumor	573 (10.49)%	376 (11.18)%	415 (2.85)%
Moderate or severe liver disease	66 (1.21)%	40 (1.19)%	17 (0.12)%
Metastatic cancer	171 (3.13)%	115 (3.42)%	64 (0.44)%
AIDS/HIV	142 (2.6)%	60 (1.78)%	63 (0.43)%
Outpatient Encounter_Count Mean (SD)	8.58 (11.74)	7.97 (10.78)	11.4(13.98)
Inpatient Encounter_Count Mean (SD)	0.28 (0.97)	0.19 (0.75)	0.04(0.23)
Same provider twice	667 (12.21)%	438 (13.02)%	5191 (35.64)%
Same provider ≥ 3	4618 (84.55)%	2973 (88.38)%	9039 (62.05)%
Influenza vaccine	247 (4.52)%	157 (4.67)%	0 (0)%
Pneumococcal vaccine	90 (1.65)%	32 (0.95)%	0 (0)%
Mammography	206 (3.77)%	306 (9.1)%	941 (6.46)%
Pap smear	36 (0.66)%	24 (0.71)%	3402 (23.35)%
Colonoscopy	268 (4.91)%	191 (5.68)%	1876 (12.88)%
PSA test	45 (0.82)%	44 (1.31)%	127 (0.87)%
Fecal occult blood test	89 (1.63)%	51 (1.52)%	23 (0.16)%
General medical exam	624 (11.42)%	447 (13.29)%	154 (1.06)%
HEPS category			
0-<10%	3821 (45.6%)		3860 (26.5%)
10-<25%	2275 (27.1%)		3773 (25.9%)
25-<50%	1612 (19.2%)		3715 (25.5%)
50-<75%	396 (4.7%)		1923 (13.2%)
75-100%	281 (3.4%)		1296 (8.9%)

Abbreviations: CCI, Charlson Comorbidity Index; HEPS, Harmonized Encounter Proportion Score; LABlue, Louisiana Blue; NHB, non-Hispanic Black; NHW, non-Hispanic White; PSA, prostate-specific antigen; REACHnet, Research Action for Health Network.

**Figure 2.**
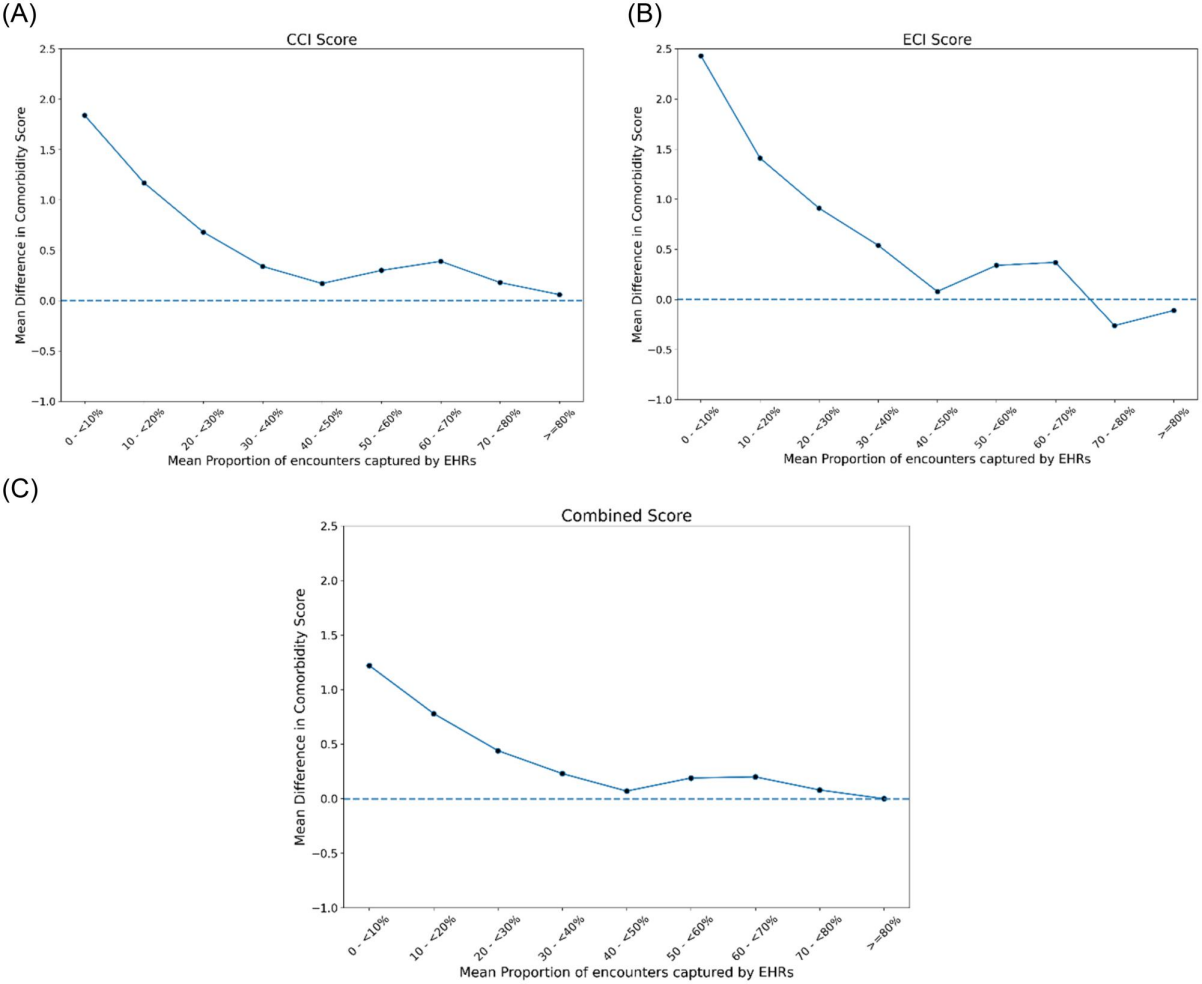
Mean difference by HEPS score deciles in (A) CCI score, (B) ECI score, and (C) Combined score in OneFlorida+. Abbreviations: CCI, Charlson Comorbidity Index; ECI, Elixhauser Comorbidity Index.

**Figure 3.**
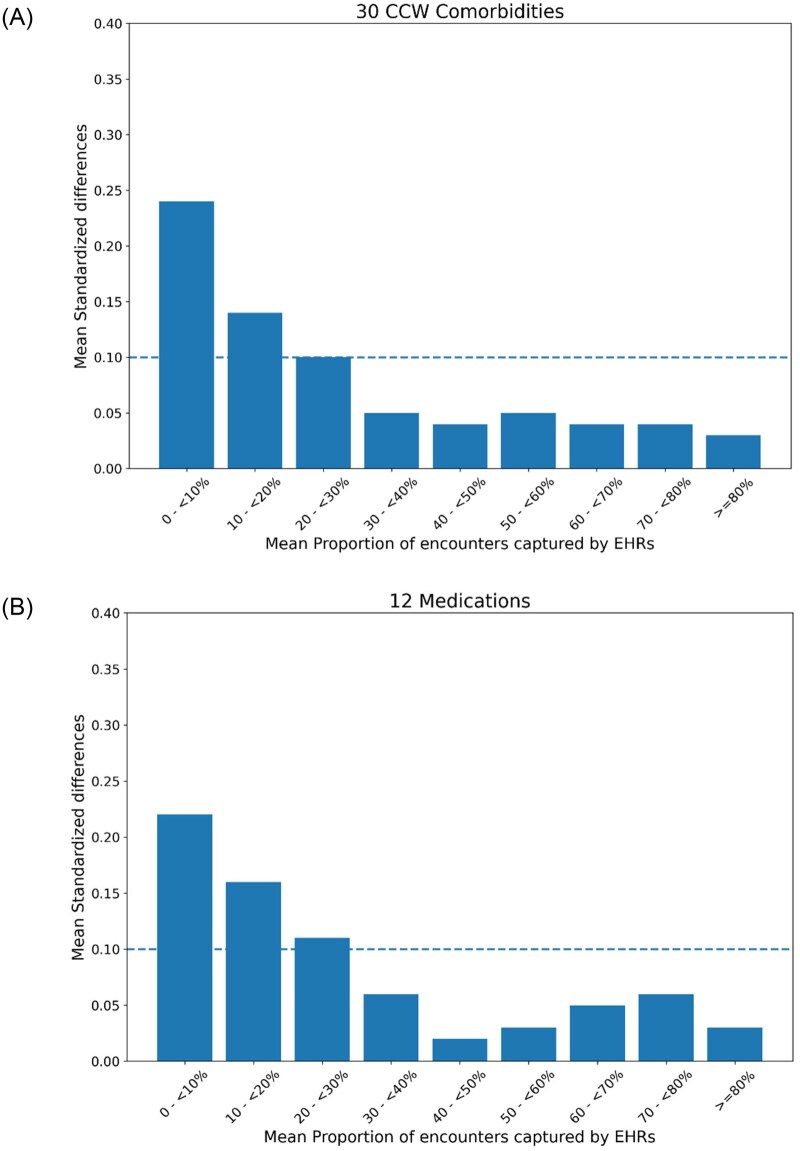
Mean standardized difference between EHR-claims linked vs EHR-only data for (A) comorbidities and (B) medications in OneFlorida+.

As shown in Figure S1A, 91.9% of patients had HEPS scores below 50%, with a mean HEPS of 20%. In Figure 2, the mean differences in comorbidity scores between EHR-only data and linked EHR-claims data were markedly lower at higher HEPS levels, reaching relatively low levels by approximately 30% and remaining low overall thereafter, despite minor fluctuations in some higher HEPS strata. For patients with HEPS < 10%, mean differences in CCI, ECI, and the combined score were 2.24, 3.14, and 1.46, which were 6.6-, 28.5-, and 9.7-fold higher, respectively, than those with HEPS ≥80% (0.34, 0.11, and 0.15). Full results with 95% confidence intervals across HEPS deciles are reported in Table S5. A similar pattern was observed in the classification of the 42 selected clinical variables. Figure 3 shows that MSDs comparing EHR-only with linked EHR-claims data were substantially reduced as HEPS increased, fell below the prespecified 0.10 benchmark at approximately 30%, and remained low overall thereafter, with minor variation across higher HEPS categories. Among patients with HEPS < 10%, the average MSDs were 0.29 for comorbidity variables and 0.32 for medication variables—7.25-fold and 4-fold higher, respectively, than MSDs in patients with HEPS ≥80% (0.04 and 0.08). Full MSD results and confidence intervals by decile are provided in Table S6. Using MSD of 0.10 as the cutoff,^24^^,^^25^ a minimum HEPS threshold of 30% was necessary to achieve acceptable classification of the selected variables in OneFlorida+, ensuring standardized differences were below the predefined cutoff for misclassification. Accordingly, we defined patients with HEPS ≥30% as the “EHR high-continuity cohort.” Sensitivity analysis, presented in Figure S2, further confirmed this pattern, showing a consistent increase in the average sensitivity of EHRs in capturing codes for the 42 selected variables as HEPS increased, validating HEPS as a strong proxy for data completeness. Lastly, we examined the distribution of data continuity across racial and ethnic groups (Table 2). The NHW subgroup had a higher proportion of patients with high EHR continuity compared with the NHB and other racial/ethnic groups. Both NHW and NHB patients were more frequently represented in the high-continuity group than in the low-continuity group, although the disparity remained more pronounced for NHW.

**Table 2.** Number of patients and their distribution by high vs low data-continuity in racial subgroups in OneFlorida+.

EHR data-continuity	NHW	NHB	Hispanic	Unknown	Others
High, *N* = 2007	884 (44.05%)	682 (33.98%)	376 (18.73%)	44 (2.19%)	21 (1.05%)
Low, *N* = 6378	2611 (40.94%)	1779 (27.89%)	1814 (28.44%)	135 (2.12%)	39 (0.61%)

Abbreviations: EHR, electronic health record; NHB, non-Hispanic Black; NHW, non-Hispanic White.

While both LR and XGBoost continuity-prediction algorithms demonstrated excellent performance in identifying patients with high EHR continuity in both internal and external validation (Table 3), XGBoost consistently outperformed LR across all predictor sets. Models trained with the complete predictor set achieved the best performance, with improved AUROC and F1-score compared with models trained on predictor set 1 identified from Huang et al’s^20^ paper or on predictor set 2 identified from Lin et al’s paper.^8^ Overall, the XGBoost model using combined data showed the best predictive performance, achieving an AUROC of 0.77.

**Table 3.** Comparative performance of the models in OneFlorida+.

Data	Model	AUROC	F1-score	Specificity	Accuracy
Complete predictor set	XGBoost	0.77	0.54	0.72	0.71
	LR	0.75	0.53	0.68	0.68
Predictor set 1	XGBoost	0.77	0.53	0.73	0.71
	LR	0.73	0.51	0.67	0.67
Predictor set 2	XGBoost	0.73	0.51	0.72	0.69
	LR	0.72	0.51	0.69	0.68

Abbreviations: AUROC, area under the receiver operating characteristic curve; LR, logistic regression.

**Figure 4.**
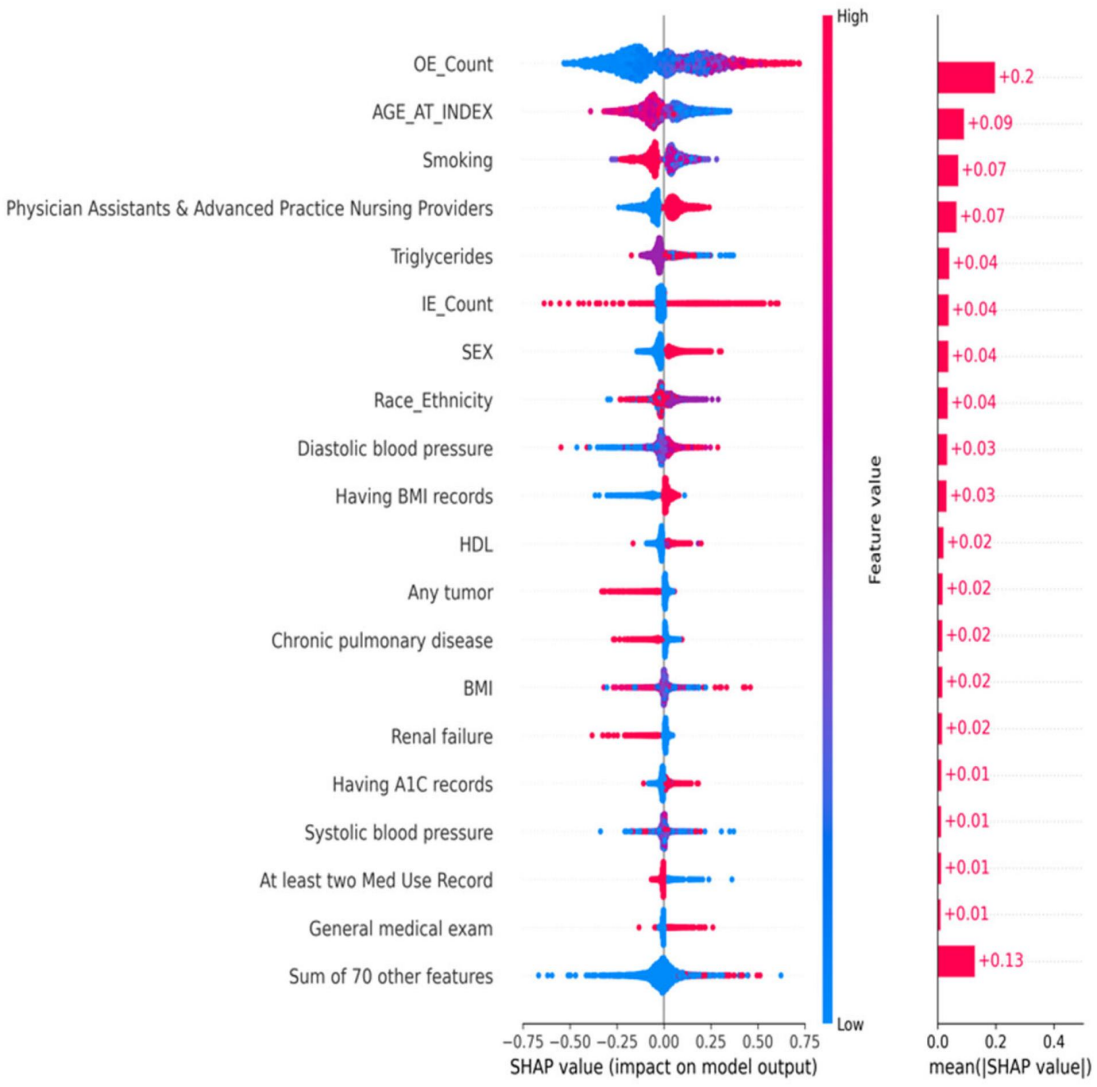
SHAP values for XGBoost on the complete predictor set, illustrating the relative contribution of each predictor to the probability of high EHR care continuity.

Subsequently, using the XGBoost model with the complete predictor set, we assessed model interpretability with SHAP values (Figure 4) to identify key predictors of high EHR continuity. In Figure 4, sex and smoking were displayed after post hoc combination of one-hot encoded categories; thus, Sex can be interpreted as female vs male (higher values indicating male), whereas Smoking was combined across current, former, never, and unknown categories, with the SHAP pattern suggesting that unknown smoking status tended to contribute more negatively to prediction of high EHR continuity, while defined smoking categories more often contributed positively. These patterns likely reflect documentation and data-capture signals rather than a true ordinal effect. Significant predictors included features from both data sources, such as “outpatient encounter count,” “triglycerides,” and “having body mass index (BMI) records.” These findings highlight the importance of incorporating clinical and encounter-related variables in predictive modeling for EHR continuity. The SHAP analyses for LR and additional datasets are provided in Figures S3-S7. We further evaluated model fairness by examining the FNR across racial and ethnic subgroups in the XGBoost models (Table 4), with additional metrics available in Table S7. Overall, the algorithm appeared biased against the Hispanic group relative to NHW; fairness metrics improved significantly after applying the adversarial debiasing technique.

**Table 4.** FNR across race-ethnicity groups in XGBoost models.

		NHB (protected) and NHW		Hispanic (protected) and NHW	
		Protected_FNR	Privileged_FNR	Protected_FNR	Privileged_FNR
Complete predictor set	Original	0.25	0.35	0.60	0.35
		0.71		1.69	
	After mitigation	0.50	0.53	0.46	0.50
		0.93		0.92	
Predictor set 1	Original	0.26	0.35	0.60	0.36
		0.72		1.69	
	After mitigation	0.50	0.55	0.46	0.51
		0.91		0.91	
Predictor set 2	Original	0.33	0.35	0.52	0.35
		0.96		1.51	
	After mitigation	0.33	0.41	0.25	0.33
		0.81		0.75	

Abbreviations: FNR, false negative rate; NHB, non-Hispanic Black; NHW, non-Hispanic White.

In the external validation using REACHnet data, 77.9% of patients had HEPS < 50% (mean 31%; Figure S1B). Mean differences between EHR-only and linked EHR-claims comorbidity scores declined with increasing HEPS and stabilized at around 30% (Figure S4); a consistent pattern was observed for the 42 variables, with MSD decreasing as HEPS increased (Figure S5). Because MSDs for the 12 medication variables were uniformly small (<0.05), the 30 condition variables informed threshold selection; applying the prespecified MSD < 0.10 criterion, HEPS ≥30% was required for acceptable classification, defining the “EHR high-continuity cohort.” By race/ethnicity groups (Table S8), the Hispanic subgroup had the highest high-continuity proportion, whereas NHB and other groups had < 20%. Model performance was stable across predictor sets (Table S9): predictor set 1 showed the strongest transportability (XGBoost AUROC 0.81, F1 0.75; LR AUROC 0.78, F1 0.73), the complete predictor set offered similar discrimination (AUROC 0.74-0.76) with XGBoost maintaining higher specificity and accuracy than LR (0.71/0.68 vs 0.41/0.65), and predictor set 2 yielded lower performance (LR AUROC 0.76; XGBoost AUROC 0.65 with higher specificity 0.76).

## Discussion

Using linked EHR-claims data, we quantified misclassification across comorbidity indices and 42 clinical variables and identified a pragmatic threshold (HEPS ≥30%) defining an “EHR high-continuity cohort.” We then developed person-level continuity classifiers that achieved excellent discrimination in internal testing (best AUROC ≈0.77) and transported well to an independent network.

In this study, we evaluated the magnitude of completeness of patient medical information captured in the OneFlorida+ EHR system by estimating EHR data continuity using a HEPS approach, a data continuity—measuring index adapted from prior MPEC algorithm.^8^^,^^10^ Our results showed that the mean HEPS in the study cohort was 20%, with the 91.9% of patients having HEPS values below 50%. These findings were consistent with previous studies reporting mean MPEC of 27% and 26% in Massachusetts and North Carolina EHR systems, with 74% and 78% of patients having MPEC below 50%.^10^ Taken together, these estimates suggest a single network does usually not comprehensively capture all clinically relevant encounters for most patients in the cohort. Despite the relatively low HEPS distribution, we found that a HEPS threshold of 30% was sufficient to achieve acceptable low misclassification (MSD < 0.10) for 42 commonly used comorbidity and medication variables. This threshold was further supported by the sensitivity analyses and mean difference analyses of comorbidity scores; although small fluctuations were observed in some higher HEPS strata in OneFlorida, disagreement was substantially reduced by approximately 30% and remained low overall thereafter. We interpret these fluctuations as local variation across adjacent HEPS strata rather than a contradiction of the broader pattern. Consistent patterns were observed in external validation using the REACHnet-LABlue dataset, where the mean HEPS was 30% and misclassification decreased monotonically with increasing HEPS. These findings suggest that HEPS offer a practical approach for researchers to quantify the extent of information bias associated with low EHR continuity and to assess the potential impact of data incompleteness on study validity.

Using OneFlorida+ data, we developed and validated ML models to predict high EHR continuity. Although all models performed reasonably well, the complete predictor set performed slightly better overall than the more limited literature-based sets, supporting the value of integrating broader EHR information beyond predefined proxy indicators. External validation in REACHnet-LABlue further supported the feasibility of this approach for improving cohort selection and reducing information bias in real-world EHR research.

Some leading predictors, such as outpatient encounter counts and BMI or A1C documentation, may partly reflect EHR data density rather than continuity alone. However, because the outcome was defined by HEPS against linked claims, these variables can be interpreted as practical proxies for whether care was captured within the indexed EHR system. The model therefore is not designed to distinguish with certainty between a patient with low continuity and a patient who is simply healthy and has low healthcare utilization. Rather, it identifies whether the available EHR data appear sufficiently complete for research use. In this context, sparse EHR data may reflect either low healthcare utilization or substantial out-of-system care, both of which can limit complete ascertainment in EHR-based research. The SHAP analyses also suggested that older age was associated with lower predicted HEPS.

These findings also suggest potential strategies to mitigate data discontinuity. Features such as outpatient encounter history and documentation of BMI, HbA1c, and smoking status may identify domains where improving structured capture and longitudinal follow-up could strengthen data completeness. In practice, health systems may improve continuity not only through broader data linkage and health information exchange, but also by targeting more consistent recording of routinely collected clinical variables, especially for patients with sparse utilization or fragmented care. Thus, the SHAP results are informative not only for prediction, but also for identifying documentation and care-delivery processes that may be leveraged to improve EHR data integrity.

More broadly, continuity prediction models may also have operational and clinical applications. Health systems could use HEPS or similar measures to identify patients whose fragmented care may lead to gaps in chronic disease management, medication reconciliation, or preventive screening. Integrating continuity scores into population health dashboards or risk stratification tools could help care managers proactively engage patients likely to have incomplete EHR documentation or inconsistent follow-up. From a research perspective, embedding continuity-aware cohort selection into real-world evidence pipelines may enhance both study rigor and fairness by ensuring equitable representation of patients with reliable data capture. However, improved algorithmic fairness does not address the structural drivers of differential data completeness across groups. Adversarial debiasing reduces subgroup disparities in model performance, but not the underlying causes of EHR fragmentation, such as unequal access or out-of-system care. It should therefore be viewed as a strategy to improve predictive parity and reduce disproportionate exclusion, rather than as a solution to inequitable data capture.

This study has several limitations that inform next steps. First, because HEPS relies on linked EHR and claims data, it reflects continuity within observable data rather than complete healthcare utilization. Some encounters may remain unobserved, including out-of-network, uninsured, self-pay, and other non-captured services. In addition, patients whose EHR records cannot be linked to claims may also differ systematically in insurance coverage and care-seeking patterns, which may not be randomly distributed across demographic groups. Accordingly, although claims data were used as the reference standard for healthcare utilization in this study, they should be interpreted as the best available operational benchmark rather than a perfect gold standard. Second, both model development and evaluation were limited to data from Florida and Louisiana. The EHR continuity prediction model was developed using EHR-Medicaid claims–linked data and externally validated in EHR-commercial claims–linked data. While validation in an independent state-based dataset supports preliminary transportability, differences in demographic, clinical, and utilization patterns across healthcare systems may affect model calibration and operating characteristics, and the extent to which the model generalizes to older Medicare populations or other health systems remains unclear. This is particularly important because the SHAP analyses linked older age to lower predicted HEPS, possibly reflecting more distributed care and lower observability within a single EHR network. A similar concern applies in safety-net settings, where uninsured or non-linkable care may be more common and more strongly correlated with ethnicity; in such contexts, low predicted continuity may reflect limited observability of care rather than fragmentation alone. Future work should examine model performance, calibration, and fairness across broader geographic regions and more heterogeneous care settings, with local recalibration considered as needed prior to implementation.

## Conclusion

In conclusion, we developed a fair, generalizable ML model that exhibits excellent predictive performance in identifying patients with high EHR continuity. These findings underscore the potential of our model to enhance the quality of EHR data for research by accurately identifying individuals with sufficient and reliable clinical documentation.

## Supplementary Material

ooag124_Supplementary_Data

## Data Availability

The data underlying this article cannot be shared publicly due to privacy regulations, Institutional Review Board restrictions, and data use agreements with OneFlorida+, REACHnet, LA Blue, and the Centers for Medicare & Medicaid Services (Medicare claims data). The data will be shared on reasonable request to the corresponding author, subject to approval by the relevant data custodians and ethics committees.
